# Associations Between Serum 25-Hydroxyvitamin D Levels and Metabolic Syndrome Among Korean Adolescents: Based on the Korea National Health and Nutrition Examination Survey in 2022–2023

**DOI:** 10.3390/nu18020360

**Published:** 2026-01-22

**Authors:** Min Hyung Cho, Young Suk Shim, Hae Sang Lee

**Affiliations:** Department of Pediatrics, Ajou University Hospital, School of Meidicine, Ajou University, 164 World Cup-ro, Yeongtong-gu, Suwon 16499, Republic of Korea; chomh89@naver.com (M.H.C.); royjays@gmail.com (Y.S.S.)

**Keywords:** vitamin D, 25-hydroxyvitamin D, metabolic syndrome, central obesity, Korea national health and nutrition examination survey

## Abstract

**Background/Objectives:** Vitamin D is a nutrient involved not only in bone metabolism but also in metabolic functions, and deficiency is common during adolescence. This study aimed to describe the distribution of serum 25-hydroxyvitamin D levels among Korean adolescents and to examine their associations with metabolic syndrome and its individual components. **Methods:** We analyzed data from the 2022–2023 Korea National Health and Nutrition Examination Survey. Adolescents aged 10–18 years with serum 25-hydroxyvitamin D measurements were included (unweighted N = 880). Weighted analyses were performed by categorizing serum 25-hydroxyvitamin D levels into quartiles. Associations between vitamin D quartiles and anthropometric and metabolic parameters were examined using complex-sample general linear models, and odds ratios for metabolic syndrome and its individual components according to vitamin D deficiency were estimated using complex-sample logistic regression models. **Results:** Weighted prevalence of vitamin D deficiency (<20 ng/mL) was 62.4%, higher in females than males. Higher 25(OH)D quartiles were inversely associated with obesity-related indices, including BMI, waist circumference, and waist-to-height ratio, after full adjustment (*p* for trend < 0.05). No significant associations were observed for blood pressure, fasting glucose, or lipid parameters. In dichotomous analyses (<20 vs. ≥20 ng/mL), vitamin D deficiency was associated with higher odds of waist circumference ≥ 90th percentile (OR 2.59), waist-to-height ratio > 0.5 (OR 2.63), and BMI ≥ 95th percentile (OR 1.89), while metabolic syndrome was not significant. **Conclusions:** Vitamin D appears to play an important role in metabolic health in adolescents and was particularly associated with general and central obesity.

## 1. Introduction

Vitamin D is an essential fat-soluble nutrient that plays a key role in calcium and phosphorus homeostasis and skeletal mineralization [1,2]. Beyond its role in bone health, vitamin D has been associated with extra-skeletal functions, such as immune regulation, glucose metabolism, and cardiovascular health [3,4]. Despite its importance, vitamin D insufficiency is highly prevalent among children and adolescents, particularly in Asian populations, largely due to limited sunlight exposure, indoor lifestyles, and dietary inadequacy [5,6]. Metabolic syndrome, defined by a cluster of cardiometabolic abnormalities such as central obesity, dyslipidemia, elevated blood pressure, and impaired glucose regulation, was traditionally considered an adult condition but has become increasingly prevalent among children and adolescents in parallel with the global rise in pediatric obesity [7].

Previous studies have examined the association between vitamin D status and metabolic syndrome, primarily in adult populations, with many reporting inverse relationships between serum 25-hydroxyvitamin D [25(OH)D] levels and the prevalence of metabolic syndrome or its individual components, including central obesity, dyslipidemia, insulin resistance, and elevated blood pressure [8,9,10]. However, because metabolic syndrome is not routinely assessed and serum 25(OH)D is not commonly measured in adolescents, evidence in this age group has largely relied on cross-sectional analyses of population-based survey data, including the Korea National Health and Nutrition Examination Survey (KNHANES) [11,12]. However, because serum 25(OH)D was not assessed in the KNHANES from 2014 to 2021, few studies have examined the association between vitamin D status and metabolic syndrome among Korean adolescents during this period.

This study aimed to investigate the association between vitamin D status and metabolic syndrome and its components among Korean adolescents using data from the 2022–2023 KNHANES.

## 2. Materials and Methods

### 2.1. Subjects

The Korea National Health and Nutrition Examination Survey (KNHANES) is a nationwide, population-based cross-sectional survey conducted by the Korea Disease Control and Prevention Agency [13]. Initiated in 1998, KNHANES has been performed annually using a complex, multistage, stratified probability sampling design to obtain nationally representative data on the health and nutritional status of the non-institutionalized Korean population aged 1 year and older. The survey consists of health interviews, health examinations, and nutrition assessments [14].

**Figure 1:**
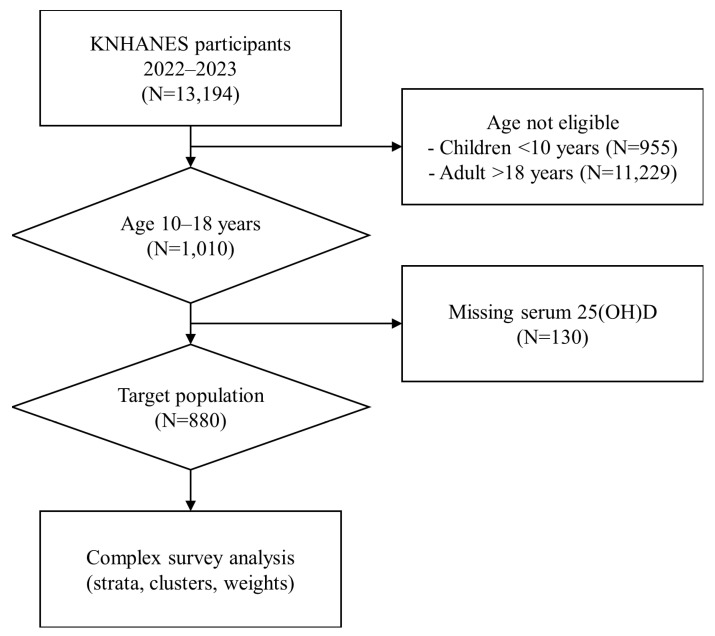
Flow diagram of participant selection from the Korea National Health and Nutrition Examination Survey (KNHANES) 2022–2023. 25(OH)D, 25-hydroxyvitamin D.

Following the resumption of serum 25-hydroxyvitamin D [25(OH)D] measurement in the Korea National Health and Nutrition Examination Survey after its suspension since 2014, a total of 13,194 participants from the 2022–2023 survey cycles were initially considered. For the purpose of this study, analyses were restricted to adolescents aged 10–18 years, resulting in the identification of 1080 eligible participants. Among them, 130 individuals with missing serum 25(OH)D data were excluded. Consequently, data from a final sample of 880 adolescents were included in the analysis (Figure 1).

This study was conducted in accordance with the Declaration of Helsinki. The Institutional Review Board of the Korea Disease Control and Prevention Agency approved the KNHANES data collection procedures, and written informed consent was obtained from all participants. As this study involved secondary analysis of anonymized KNHANES data, ethical review and approval were waived by the Institutional Review Board of Ajou University Hospital.

### 2.2. Anthropometrics and Measurements

Height, weight, and waist circumference were measured in all participants. Height and weight were measured to the nearest 0.1 cm and 0.1 kg, respectively, using standardized procedures. Body mass index (BMI) was calculated as weight divided by height squared (kg/m^2^). Age- and sex-specific standard deviation scores (SDSs) for height, weight, and BMI were calculated using the mean and standard deviation values from the 2017 Korean National Growth Charts [15]. Waist circumference was measured at the narrowest point between the lower margin of the rib cage and the iliac crest. Because waist circumference reference values are not provided in the 2017 Korean National Growth Charts, sex-specific reference data derived from a Korean population were used for standardization [16].

Blood pressure was measured up to three times during a single visit. The average of the second and third systolic and diastolic blood pressure measurements was used for analysis. Blood pressure status was classified according to age-, sex-, and height-specific percentiles based on the American Academy of Pediatrics clinical practice guideline [17]. Average sleep duration was calculated as the mean of weekday and weekend sleep times. Daily sedentary time was assessed using questionnaire data. Lifetime alcohol consumption, engagement in at least 60 min of physical activity on one or more days per week, and participation in muscle-strengthening exercise at least once per week were evaluated based on self-reported questionnaires.

Serum 25(OH)D concentrations were measured using radioimmunoassay (RIA) until 2014. In the 2022–2023 KNHANES cycles, vitamin D levels were measured using liquid chromatography–tandem mass spectrometry (LC-MS). For descriptive analyses, vitamin D status was categorized according to conventional cut-off values as deficiency (<20 ng/mL), insufficiency (20–29 ng/mL), and sufficiency (≥30 ng/mL), and the weighted prevalence of each category was estimated. For analytic purposes, the associations between serum 25(OH)D and metabolic outcomes were additionally examined by categorizing 25(OH)D levels into quartiles and into two groups (<20 ng/mL vs. ≥20 ng/mL). Metabolic parameters were assessed under fasting conditions after the evening meal on the previous day and included fasting plasma glucose, total cholesterol, triglycerides, high-density lipoprotein cholesterol (HDL), and low-density lipoprotein cholesterol (LDL). From 2022 onward, low-density lipoprotein cholesterol was determined by direct measurement rather than by a calculation-based method.

### 2.3. Definition of Metabolic Syndrome

Metabolic syndrome was defined according to the modified National Cholesterol Education Program Adult Treatment Panel III (NCEP-ATP III) criteria [18]. The diagnostic components included triglyceride levels ≥ 110 mg/dL, high-density lipoprotein cholesterol ≤ 40 mg/dL, waist circumference at or above the 90th percentile for age and sex, fasting plasma glucose ≥ 110 mg/dL, and systolic or diastolic blood pressure at or above the 90th percentile for age, sex, and height. Metabolic syndrome was diagnosed when three or more of these criteria were met. Participants receiving antihypertensive, lipid-lowering, or antidiabetic medications were considered to meet the corresponding criteria regardless of the measured values. In addition, obesity-related factors were assessed. General obesity was defined as a body mass index at or above the 95th percentile for age and sex. Abdominal obesity was defined as a waist-to-height ratio of 0.5 or higher, calculated as waist circumference divided by height [19].

### 2.4. Statistical Analysis

All statistical analyses were performed using SPSS statistics version 29 (IBM Corp., Armonk, NY, USA). Because the Korea National Health and Nutrition Examination Survey employs a multistage, stratified cluster sampling design, participants within the same primary sampling unit are not statistically independent, and standard analyses assuming simple random sampling would underestimate standard errors and inflate type I error rates. Therefore, all analyses were conducted using the complex samples procedures, incorporating sampling strata, primary sampling units, and examination weights through a complex sample plan file, with variances estimated using Taylor series linearization, which derives standard errors from the variability between primary sampling units rather than treating all individuals as independent observations.

To examine trends in metabolic and anthropometric variables according to vitamin D status, participants were categorized into quartiles based on serum 25(OH)D levels. Linear trends across increasing vitamin D quartiles were assessed by modeling the quartile variable as an ordinal continuous variable using complex samples multivariable linear regression. The regression coefficient (B) and the corresponding *p* value for trend were calculated. In addition, serum 25(OH)D levels were classified into two groups based on a cutoff value of 20 ng/mL to examine their associations with obesity and components of metabolic syndrome. Odds ratios and corresponding *p* values were estimated using complex samples multivariable logistic regression. Two models were analyzed. Model 1 was adjusted for age and sex. Model 2 was additionally adjusted for sleep duration, sedentary time, alcohol consumption, physical activity, and muscle-strengthening exercise.

For all subgroup analyses, the full analytic dataset was retained and domain analysis was applied within the complex samples framework rather than creating separate subset files, in order to preserve the original survey design and obtain valid variance estimates. Missing or invalid responses were excluded according to the KNHANES coding scheme, and only observations with valid values for the variables included in each model were analyzed. A *p* value of less than 0.05 was considered statistically significant.

## 3. Results

**Figure 2:**
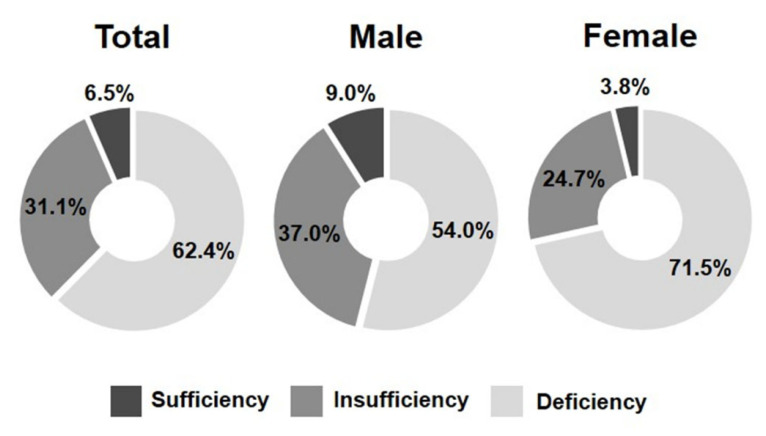
Weighted distribution of serum 25-hydroxyvitamin D status (Deficiency, <20 ng/mL; Insufficiency, 20–29 ng/mL; Sufficiency ≥ 30 ng/mL) among Korean adolescents overall and stratified by sex.

A total of 880 adolescents were included in the analysis based on unweighted counts, comprising 457 males (51.9%) and 423 females (48.1%). When weighted to represent the national adolescent population, 6.5% were classified as having sufficient serum 25(OH)D levels (≥30 ng/mL), 31.1% as insufficient (20–29 ng/mL), and the remaining 62.4% as deficient (<20 ng/mL). When stratified by sex, vitamin D deficiency was more prevalent among females (71.5%) than males (54.0%), whereas vitamin D sufficiency was more common in males (9.0%) than in females (3.8%) (Table 1, Figure 2).

When serum 25(OH)D levels were categorized into quartiles (Q1–Q4), differences in anthropometric, lifestyle, and metabolic characteristics across quartiles were examined. Lower serum 25(OH)D levels were observed in younger participants and in females compared with males. Therefore, Model 1 was adjusted for age and sex to assess linear trends across increasing 25(OH)D quartiles. In age- and sex-adjusted analyses (Model 1), significant inverse linear trends were observed across increasing serum 25(OH)D quartiles for multiple anthropometric parameters, including height standard deviation score (SDS), body weight, weight SDS, body mass index (BMI), BMI SDS, waist circumference, and waist-to-height ratio (all *p* for trend < 0.05). In contrast, no significant linear trends were identified for laboratory metabolic parameters, including blood pressure, fasting plasma glucose, total cholesterol, triglycerides, high-density lipoprotein (HDL) cholesterol, or low-density lipoprotein (LDL) cholesterol. Among lifestyle-related variables, the proportion of adolescents engaging in muscle-strengthening exercise at least once per week increased significantly across higher 25(OH)D quartiles (*p* for trend = 0.022), whereas no significant trends were observed for overall physical activity, sleep duration, sedentary time, or alcohol consumption (Table 1).

In contrast, when lifestyle-related factors were additionally adjusted together with age and sex (Model 2), significant associations persisted for selected anthropometric parameters. In this fully adjusted model, higher serum 25(OH)D quartiles remained inversely associated with body weight, weight SDS, BMI, BMI SDS, waist circumference, and waist-to-height ratio (all *p* for trend < 0.05), whereas the association with height SDS was attenuated and no longer statistically significant. Among lifestyle variables, only muscle-strengthening exercise performed at least once per week continued to show a significant positive trend across increasing 25(OH)D quartiles (*p* for trend = 0.022). No significant linear trends were observed for blood pressure, fasting plasma glucose, lipid profiles, sleep duration, sedentary time, overall physical activity, or alcohol consumption after full adjustment (Table 1).

*p* Values for sex-specific differences and unadjusted regression coefficients with corresponding *p* values for linear trends across serum 25(OH)D quartiles are provided in Supplementary Table S1.

**Figure 3:**
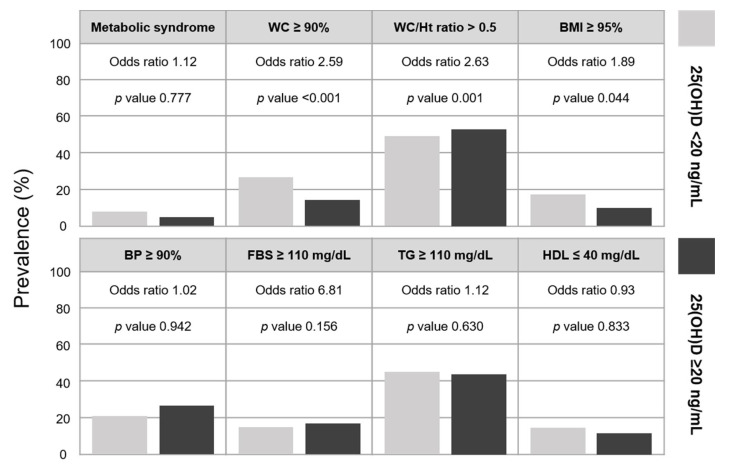
Prevalence and odds ratios of metabolic syndrome and its components according to vitamin D deficiency status. Odds ratios were calculated using complex survey logistic regression analyses. WC, waist circumference; WC/Ht ratio, waist-to-height ratio; BMI, body mass index; BP, blood pressure; FBS, fasting blood sugar; TG, triglyceride; HDL, high-density lipoprotein cholesterol; 25(OH)D, 25-hydroxyvitamin D.

To evaluate the association between serum 25(OH)D status and metabolic syndrome and its individual components, participants were categorized into two groups based on a cutoff value of 20 ng/mL, representing vitamin D deficiency (<20 ng/mL) and insufficiency/sufficiency (≥20 ng/mL). The weighted prevalence and adjusted odds ratios were summarized in Table 2 and Figure 3. The weighted prevalence of metabolic syndrome was 7.7% in the vitamin D-deficient group and 5.3% in the group with serum 25(OH)D levels ≥ 20 ng/mL. However, no statistically significant association between vitamin D status and metabolic syndrome was observed in either Model 1 or Model 2. When individual components were examined, Model 1 demonstrated significantly higher odds of central and general obesity among adolescents with vitamin D deficiency. Specifically, the odds of waist circumference ≥ 90th percentile, waist-to-height ratio > 0.5, and BMI ≥ 95th percentile were significantly higher in the vitamin D-deficient group than in the reference group. These associations remained statistically significant after further adjustment for lifestyle-related factors in Model 2, with no substantial change in the direction or magnitude of the associations (Table 2 and Figure 3).

## 4. Discussion

Using nationally representative data from the 2022–2023 Korea National Health and Nutrition Examination Survey (KNHANES), this study found that serum 25-hydroxyvitamin D [25(OH)D] levels were primarily associated with general and central obesity rather than with all components of metabolic syndrome in Korean adolescents. Lower 25(OH)D levels were associated with higher body weight, body mass index, waist circumference, and waist-to-height ratio, whereas no significant association was observed with metabolic syndrome as a composite outcome. Although vitamin D status did not influence all metabolic components, vitamin D deficiency was associated with significantly increased odds of abdominal obesity-related indicators, even after adjustment for age, sex, and lifestyle-related factors.

Vitamin D is essential for skeletal development in children through its regulation of calcium and phosphorus homeostasis [1,2]. Beyond bone health, vitamin D exerts diverse biological effects across multiple organ systems, highlighting its importance during childhood and adolescence [20,21]. Nevertheless, several studies have reported that serum vitamin D levels in children and adolescents are generally lower than those in adults [5,6]. In our study, the mean serum 25(OH)D concentration among adolescents was 18.80 ± 0.34 ng/mL, substantially lower than that observed in adults (23.46 ± 0.18 ng/mL). Using conventional cutoff values of 20 and 30 ng/mL, only 6.5% of adolescents were classified as vitamin D sufficient, while more than half had levels consistent with vitamin D deficiency, with an even lower prevalence of sufficiency among girls (3.8%). In addition, when serum vitamin D levels were categorized into quartiles, the cutoff separating Q3 and Q4 was 22.84 ng/mL, indicating that approximately three-quarters of the study population (Q1–Q3) had serum 25(OH)D levels within the deficient or near-deficient range.

Although there is ongoing debate regarding the optimal cutoff values for defining adequate vitamin D status in children and adolescents, serum 25(OH)D concentrations among Korean adolescents remain generally low [22,23]. Nevertheless, when compared with previous studies based on KNHANES data collected before 2014, despite differences in assay methods, the proportion of adolescents classified as vitamin D sufficient has modestly increased, accompanied by a corresponding decrease in the prevalence of deficiency [24,25]. In addition, prior studies that similarly categorized vitamin D status into quartiles reported lower cutoff values for the upper quartiles than those observed in the present study, suggesting an overall upward shift in serum 25(OH)D concentrations over time [11]. This trend may, at least in part, reflect increased awareness of the importance of vitamin D, greater engagement in outdoor activities, and the growing popularity of vitamin D supplementation in Korea.

When serum 25(OH)D levels were analyzed in quartiles, significant inverse linear trends were observed between vitamin D status and multiple obesity-related indices. These findings are consistent with those reported in large-scale population-based surveys conducted in various countries, as well as with results from meta-analyses [5,12,26,27]. Although BMI is widely used as an indicator of general adiposity, it does not adequately capture body fat distribution or visceral adiposity [28]. In children and adolescents, the waist-to-height ratio is considered particularly useful, as it is less dependent on age- and sex-specific reference values than waist circumference alone [29]. In the present study, not only body weight and BMI but also waist circumference and waist-to-height ratio showed a significant inverse association with serum vitamin D levels. Notably, this association remained robust even after adjustment for multiple lifestyle-related factors. Furthermore, the associations of waist circumference and waist-to-height ratio with vitamin D status persisted even after additional adjustment for body weight and BMI, suggesting an independent relationship between central adiposity and vitamin D levels.

Regarding cardiometabolic outcomes such as blood pressure and lipid profiles, large population-based studies have reported inconsistent findings on their associations with vitamin D status. In a nationwide cross-sectional study conducted among children and adolescents in Iran, vitamin D deficiency was associated with low HDL cholesterol levels and elevated fasting glucose [30]. In contrast, analyses based on the United States National Health and Nutrition Examination Survey reported associations between vitamin D status and systolic blood pressure as well as HDL cholesterol [31]. Furthermore, findings from the 2008–2010 Korean national survey data demonstrated significant associations between vitamin D quartiles and systolic blood pressure, diastolic blood pressure, and fasting glucose levels [11]. However, in the present study, no significant associations were observed between vitamin D levels and blood pressure or lipid parameters, including total cholesterol, triglycerides, HDL cholesterol, and LDL cholesterol. Several factors should be considered when interpreting these findings. First, even in large-scale studies, overt cardiometabolic abnormalities such as hypertension, hyperglycemia, and dyslipidemia are relatively uncommon in pediatric populations. Second, pubertal status and related hormonal changes, which are important determinants of blood pressure and lipid levels, may not have been adequately captured in our analysis, potentially attenuating the observed associations [32]. In addition, family history is widely recognized as a major determinant of hypertension and dyslipidemia, and the lack of adjustment for familial predisposition may have contributed to the inconsistencies reported across large-scale studies [33].

Consistent findings were obtained when analyses of metabolic syndrome and its components were repeated using two vitamin D categories, defined as deficiency (<20 ng/mL) and non-deficiency (≥20 ng/mL). The estimated prevalence of metabolic syndrome was higher in the vitamin D-deficient group (7.7%) than in the non-deficient group (5.3%). However, this difference was no longer statistically significant after adjustment for age, sex, and other relevant covariates. In addition, comparisons of blood pressure, triglycerides, and fasting glucose showed no significant differences in odds ratios according to vitamin D deficiency status. However, significant associations persisted for obesity-related indices. Vitamin D deficiency was associated with higher odds of obesity (OR 1.89), waist circumference ≥90th percentile (OR 2.63), and waist-to-height ratio > 0.5 (OR 2.63) compared with the non-deficient group. Although vitamin D deficiency was not independently associated with the presence of metabolic syndrome, its strong relationship with central obesity suggests that vitamin D remains a relevant factor in the early pathophysiology of metabolic syndrome.

Our findings are broadly consistent with those of a systematic review and dose–response meta-analysis, which reported an inverse association between serum 25(OH)D levels and the risk of metabolic syndrome [34]. Hajhashemy et al. further reported that although lower vitamin D levels were associated with a higher risk of metabolic syndrome in the majority of included studies, this association was no longer statistically significant when the analysis was restricted to prospective cohort studies. It should be noted that this meta-analysis was conducted in adult populations and focused on metabolic syndrome as a composite outcome, without providing component-specific estimates for indices such as central obesity, which limits direct comparison with our findings. Moreover, Li et al. examined metabolic risk in children and adolescents using a continuous metabolic syndrome risk score based on age- and sex-specific z-scores, an approach adopted because the prevalence of metabolic syndrome is relatively low in this age group [35]. They reported a significant inverse association between serum 25(OH)D levels and overall metabolic risk, with waist circumference emerging as the most robust determinant, whereas associations with blood pressure and lipid parameters were inconsistent. These findings are consistent with our observation of a strong relationship between vitamin D deficiency and central obesity. However, Li et al. further highlighted the roles of systemic inflammation, lipid composite parameters, and indices of insulin resistance, which were not assessed in the present study, indicating the need for future research incorporating these factors.

This study has the strength of being based on a large, nationally representative dataset from the KNHANES. However, several limitations should be noted. Due to its cross-sectional design, the present analysis cannot determine the temporal sequence or whether vitamin D deficiency is a cause or a consequence of adverse metabolic profiles. In addition, important factors such as pubertal status and family history were not available. Although lifestyle-related variables, including sleep duration and physical activity, were considered, these were assessed through self-reported questionnaires, which may limit their accuracy [36,37].

## 5. Conclusions

In conclusion, although a causal relationship cannot be inferred from this cross-sectional study, vitamin D deficiency in adolescents was associated primarily with obesity, particularly central adiposity, rather than with metabolic syndrome itself. This finding suggests that assessment of serum 25(OH)D levels may provide clinically useful information in adolescents with abdominal obesity or elevated metabolic risk, and supports the need for longitudinal studies to clarify its role in the progression of metabolic syndrome.

## Figures and Tables

**Table 1:** Weighted characteristics of study participants according to serum 25-hydroxyvitamin D [25(OH)D] quartiles and linear trends assessed using complex samples multivariable regression.

			Serum 25(OH)D (ng/mL)				Model 1 *		Model 2 ^†^	
	Male	Female	Q1 (0–13.43)	Q2 (13.43–17.88)	Q3 (17.88–22.84)	Q4 (≥22.84)	B (95% CI)	*p* for Trend	B (95% CI)	*p* for Trend
N	457	423	205	211	229	235				
Age (years)	14.14 ± 0.14	13.90 ± 0.14	15.02 ± 0.19	14.19 ± 0.20	13.61 ± 0.19	13.27 ± 2.10				
Height (cm)	166.29 ± 0.67	157.66 ± 0.42	164.42 ± 0.81	162.39 ± 0.86	161.54 ± 0.87	160.11 ± 1.08	−0.36 (−0.72, 0.20)	0.209	−0.45 (−0.98, 0.08)	0.095
Height SDS	0.82 ± 0.06	0.57 ± 0.06	0.66 ± 0.09	0.71 ± 0.08	0.76 ± 0.07	0.66 ± 0.10	−0.08 (−0.16, −0.00)	0.047	−0.08 (−0.17, 0.01)	0.097
Weight (kg)	61.78 ± 1.03	51.52 ± 0.73	60.39 ± 1.28	57.83 ± 1.04	55.84 ± 1.62	53.19 ± 1.34	−1.51 (−2.46, −0.57)	0.002	−1.44 (−2.49, −0.39)	0.007
Weight SDS	0.80 ± 0.09	0.49 ± 0.07	0.79 ± 0.13	0.76 ± 0.09	0.62 ± 0.14	0.44 ± 0.09	−0.19 (−0.29, −0.09)	<0.001	−0.16 (−0.27, −0.04)	0.007
BMI (kg/m^2^)	22.05 ± 0.28	20.56 ± 0.23	22.15 ± 0.36	21.76 ± 0.30	21.03 ± 0.43	20.37 ± 0.30	−0.53 (−0.82, −0.23)	<0.001	−0.43 (−0.77, −0.09)	0.014
BMI SDS	0.48 ± 0.09	0.24 ± 0.08	0.53 ± 0.13	0.50 ± 0.10	0.31 ± 0.14	0.13 ± 0.09	−0.18 (−0.29, −0.08)	<0.001	−0.15 (−0.27, −0.03)	0.018
WC (cm)	75.34 ± 0.74	68.06 ± 0.56	73.69 ± 0.91	73.15 ± 0.82	70.67 ± 1.13	69.49 ± 0.94	−1.46 (−2.24, −0.68)	<0.001	−1.27 (−2.16, −0.39)	0.005
WC/Ht ratio	0.45 ± 0.00	0.43 ± 0.00	0.45 ± 0.01	0.45 ± 0.00	0.44 ± 0.01	0.43 ± 0.00	−0.01 (−0.01, −0.00)	<0.001	−0.01 (−0.01, −0.00)	0.020
Alcohol (%)	27.4	17.3	29.4	24.5	18.1	16.2	−0.01 (−0.06, 0.00)	0.076		
SBP (mmHg)	111.33 ± 0.56	105.03 ± 0.47	108.46 ± 0.72	108.40 ± 0.68	108.59 ± 0.84	107.43 ± 0.77	−0.56 (−1.16, 0.05)	0.070	−0.42 (−1.12, 0.27)	0.233
DBP (mmHg)	65.34 ± 0.43	64.79 ± 0.36	66.20 ± 0.48	64.89 ± 0.58	64.77 ± 0.56	64.42 ± 0.58	−0.24 (−0.73, 0.26)	0.350	0.12 (−0.47, 0.71)	0.691
Fasting glucose (mg/dL)	92.17 ± 0.44	90.57 ± 0.52	89.90 ± 0.59	92.00 ± 0.83	91.70 ± 0.61	91.98 ± 0.87	−0.05 (−0.65, 0.54)	0.857	−0.06 (−0.62, 0.51)	0.844
Total cholesterol (mg/dL)	153.69 ± 0.65	163.96 ± 1.50	161.70 ± 2.32	163.92 ± 1.97	160.88 ± 1.90	160.50 ± 2.05	−0.74 (−2.73, 1.25)	0.467	0.22 (−1.99, 2.42)	0.845
Triglyceride (mg/dL)	95.72 ± 1.33	86.41 ± 2.40	90.08 ± 4.37	88.98 ± 3.19	90.97 ± 3.41	88.60 ± 7.40	−0.28 (−6.34, 5.79)	0.929	0.98 (−5.57, 7.53)	0.767
HDL (mg/dL)	53.69 ± 0.65	58.37 ± 0.78	56.13 ± 0.95	55.54 ± 1.06	55.34 ± 1.01	56.79 ± 1.05	0.26 (−0.63, 1.14)	0.569	0.36 (−0.64, 1.35)	0.481
LDL (mg/dL)	95.72 ± 1.33	97.08 ± 1.37	95.84 ± 2.18	99.48 ± 1.64	96.22 ± 1.80	93.99 ± 1.85	−1.01 (−2.79, 0.77)	0.264	−0.27 (−2.29, 1.75)	0.796
Sleep duration (hours)	7.43 ± 0.06	7.32 ± 0.07	7.20 ± 0.10	7.37 ± 0.08	7.51 ± 0.09	7.48 ± 0.10	0.02 (−0.06, 0.11)	0.616		
Sedentary time (hours)	11.39 ± 0.17	11.69 ± 0.18	11.95 ± 0.22	11.34 ± 0.23	11.36 ± 0.25	11.42 ± 0.32	−0.07 (−0.32, 0.18)	0.602		
Physical activity (%)	51.1	37.2	40.3	42.6	47.6	48.8	0.01 (−0.03, 0.05)	0.466		
Muscle exercise (%)	62.1	27.2	35.8	44.5	44.6	60.5	0.04 (0.00, 0.07)	0.022		

* Model 1: Adjusted for age and sex. ^†^ Model 2: Adjusted for age, sex, sleep duration (average of weekday and weekend), sedentary time (hours/day), alcohol consumption (≥1 lifetime use), physical activity (≥60 min/day on ≥1 day/week), and muscle-strengthening exercise (≥1 day/week). All continuous variables are presented as weighted means ± standard deviations, and categorical variables are presented as weighted percentages (%). Abbreviations: 25(OH)D, 25-hydroxyvitamin D; Q1–Q4, quartiles of serum 25-hydroxyvitamin D; B, regression coefficient; CI, confidence interval; SDS, standard deviation score; BMI, body mass index; WC, waist circumference; Ht, height; SBP, systolic blood pressure; DBP, diastolic blood pressure; HDL, high-density lipoprotein cholesterol; LDL, low-density lipoprotein cholesterol.

**Table 2:** Weighted prevalence and odds ratios of metabolic syndrome and its components according to serum 25-hydroxyvitamin D [25(OH)D] status.

	Serum 25(OH)D		Model 1 *		Model 2 ^†^	
	<20 ng/mL	≥20 ng/mL	Odds Ratio (95% CI)	*p* Value	Odds Ratio (95% CI)	*p* Value
Metabolic syndrome	7.7%	5.3%	1.60 (0.71, 3.59)	0.251	1.12 (0.51, 2.44)	0.777
WC > 90%	26.7%	14.3%	2.61 (1.72, 3.96)	<0.001	2.59 (1.56, 4.31)	<0.001
WC/Ht ratio > 0.5	49.5%	53.3%	2.46 (1.53, 3.96)	<0.001	2.63 (1.48, 4.68)	0.001
BMI ≥ 95%	17.4%	9.8%	2.02 (1.22, 3.32)	0.006	1.89 (1.02, 3.50)	0.044
BP ≥ 90%	20.7%	26.5%	1.09 (0.75, 1.58)	0.657	1.02 (0.64, 1.61)	0.942
FBS ≥ 110	14.5%	16.7%	2.39 (0.43, 13.38)	0.322	6.81 (0.48, 97.08)	0.156
TG ≥ 110	45.1%	44.4%	1.33 (0.90, 1.97)	0.149	1.12 (0.70, 1.78)	0.630
HDL ≤ 40	14.5%	11.6%	1.34 (0.70, 2.57)	0.371	0.93 (0.49, 1.77)	0.833

* Model 1: Adjusted for age and sex. ^†^ Model 2: Adjusted for age, sex, sleep duration (average of weekday and weekend), sedentary time (hours/day), alcohol consumption (≥1 lifetime use), physical activity (≥60 min/day on ≥1 day/week), and muscle-strengthening exercise (≥1 day/week). Abbreviations: 25(OH)D, 25-hydroxyvitamin D; CI, confidence interval; WC, waist circumference; Ht, height; BMI, body mass index; BP, blood pressure; FBS, fasting blood sugar; TG, triglyceride; HDL, high-density lipoprotein cholesterol.

## Data Availability

The data used in this study are from the Korean National Health and Nutrition Examination Survey, provided by the Korean Disease Control and Prevention Agency. It is a publicly accessible database at https://knhanes.kdca.go.kr/knhanes/eng/main.do (accessed on 20 January 2026), but it requires adherence to the policies of the Korean Disease Control and Prevention Agency.

## Supplementary Materials

The following supporting information can be downloaded at: https://www.mdpi.com/article/10.3390/nu18020360/s1, Table S1: Weighted sex-specific characteristics of study participants with corresponding *p* values and unadjusted regression coefficients with *p* values for linear trends across serum 25-hydroxyvitamin D [25(OH)D] quartiles assessed using complex samples regression.

## Abbreviations

The following abbreviations are used in this manuscript

25(OH)D	25-hydroxyvitamin D
BMI	Body mass index
BP	Blood pressure
FBS	Fasting blood sugar
TG	Triglyceride
HDL	High-density lipoprotein
LDL	Low-density lipoprotein
KNHANES	Korea National Health and Nutrition Examination Survey
SDS	Standard deviation score
WC	Waist circumference
WC/Ht ratio	Waist-to-height ratio

## References

[1] Holick M.F. (2006). Resurrection of vitamin D deficiency and rickets. J. Clin. Investig..

[2] Alshahrani F., Aljohani N. (2013). Vitamin D: Deficiency, sufficiency and toxicity. Nutrients.

[3] Prietl B., Treiber G., Pieber T.R., Amrein K. (2013). Vitamin D and immune function. Nutrients.

[4] Argano C., Mirarchi L., Amodeo S., Orlando V., Torres A., Corrao S. (2023). The Role of Vitamin D and Its Molecular Bases in Insulin Resistance, Diabetes, Metabolic Syndrome, and Cardiovascular Disease: State of the Art. Int. J. Mol. Sci..

[5] Kumar J., Muntner P., Kaskel F.J., Hailpern S.M., Melamed M.L. (2009). Prevalence and associations of 25-hydroxyvitamin D deficiency in US children: NHANES 2001–2004. Pediatrics.

[6] Hilger J., Friedel A., Herr R., Rausch T., Roos F., Wahl D.A., Pierroz D.D., Weber P., Hoffmann K. (2014). A systematic review of vitamin D status in populations worldwide. Br. J. Nutr..

[7] Park S.I., Suh J., Lee H.S., Song K., Choi Y., Oh J.S., Choi H.S., Kwon A., Kim H.S., Kim J.H. (2021). Ten-Year Trends of Metabolic Syndrome Prevalence and Nutrient Intake among Korean Children and Adolescents: A Population-Based Study. Yonsei. Med. J..

[8] Lee K., Kim J. (2021). Serum vitamin D status and metabolic syndrome: A systematic review and dose-response meta-analysis. Nutr. Res. Pract..

[9] Colombo C., Fabiano V., Labati L., Loiodice M., Ceruti D., Campi I., Cuneo V., Zuccotti G., Calcaterra V. (2025). Vitamin D, adiposity, and cardiometabolic risk: Insights from a multivariable cross-sectional study. Obes. Res. Clin. Pract..

[10] Song Y., Wang L., Pittas A.G., Del Gobbo L.C., Zhang C., Manson J.E., Hu F.B. (2013). Blood 25-hydroxy vitamin D levels and incident type 2 diabetes: A meta-analysis of prospective studies. Diabetes Care.

[11] Lee D.Y., Kwon A.R., Ahn J.M., Kim Y.J., Chae H.W., Kim D.H., Kim H.S. (2015). Relationship between serum 25-hydroxyvitamin D concentration and risks of metabolic syndrome in children and adolescents from Korean National Health and Nutrition Examination survey 2008–2010. Ann. Pediatr. Endocrinol. Metab..

[12] Gao Y.X., Zhang J., Man Q., Li Y., Jia S. (2022). The association between vitamin D levels and metabolic syndrome components among metropolitan adolescent population. J. Pediatr. Endocrinol. Metab..

[13] Oh K., Kim Y., Kweon S., Kim S., Yun S., Park S., Lee Y.K., Kim Y., Park O., Jeong E.K. (2021). Korea National Health and Nutrition Examination Survey, 20th anniversary: Accomplishments and future directions. Epidemiol. Health.

[14] Kweon S., Kim Y., Jang M.J., Kim Y., Kim K., Choi S., Chun C., Khang Y.H., Oh K. (2014). Data resource profile: The Korea National Health and Nutrition Examination Survey (KNHANES). Int. J. Epidemiol..

[15] Kim J.H., Yun S., Hwang S.S., Shim J.O., Chae H.W., Lee Y.J., Lee J.H., Kim S.C., Lim D., Yang S.W. (2018). The 2017 Korean National Growth Charts for children and adolescents: Development, improvement, and prospects. Korean J. Pediatr..

[16] Lee J., Kang S.C., Kwon O., Hwang S.S., Moon J.S., Kim J. (2022). Reference Values for Waist Circumference and Waist-Height Ratio in Korean Children and Adolescents. J. Obes. Metab. Syndr..

[17] Flynn J.T., Kaelber D.C., Baker-Smith C.M., Blowey D., Carroll A.E., Daniels S.R., de Ferranti S.D., Dionne J.M., Falkner B., Flinn S.K. (2017). Clinical Practice Guideline for Screening and Management of High Blood Pressure in Children and Adolescents. Pediatrics.

[18] Cook S., Weitzman M., Auinger P., Nguyen M., Dietz W.H. (2003). Prevalence of a metabolic syndrome phenotype in adolescents: Findings from the third National Health and Nutrition Examination Survey, 1988–1994. Arch. Pediatr. Adolesc. Med..

[19] Sarna A., Porwal A., Acharya R., Ashraf S., Ramesh S., Khan N., Sinha S., Sachdev H.S. (2021). Waist circumference, waist-to-height ratio and BMI percentiles in children aged 5 to 19 years in India: A population-based study. Obes. Sci. Pract..

[20] Umar M., Sastry K.S., Chouchane A.I. (2018). Role of Vitamin D Beyond the Skeletal Function: A Review of the Molecular and Clinical Studies. Int. J. Mol. Sci..

[21] Weaver C.M., Gordon C.M., Janz K.F., Kalkwarf H.J., Lappe J.M., Lewis R., O’Karma M., Wallace T.C., Zemel B.S. (2016). The National Osteoporosis Foundation’s position statement on peak bone mass development and lifestyle factors: A systematic review and implementation recommendations. Osteoporos. Int..

[22] Corsello A., Spolidoro G.C.I., Milani G.P., Agostoni C. (2023). Vitamin D in pediatric age: Current evidence, recommendations, and misunderstandings. Front. Med..

[23] Yadav B., Gupta N., Kumar P., Sasidharan R., Purohit P., Singh K., Sharma P., Singh A. (2025). Cutoff levels of serum 25-OH-D for defining vitamin D deficiency in early infancy. Pediatr. Neonatol..

[24] Nam G.E., Kim D.H., Cho K.H., Park Y.G., Han K.D., Kim S.M., Lee S.H., Ko B.J., Kim M.J. (2014). 25-Hydroxyvitamin D insufficiency is associated with cardiometabolic risk in Korean adolescents: The 2008-2009 Korea National Health and Nutrition Examination Survey (KNHANES). Public Health Nutr..

[25] Park J.H., Hong I.Y., Chung J.W., Choi H.S. (2018). Vitamin D status in South Korean population: Seven-year trend from the KNHANES. Medicine.

[26] Fu Z., Xu C., Shu Y., Xie Z., Lu C., Mo X. (2020). Serum 25-hydroxyvitamin D is associated with obesity and metabolic parameters in US children. Public Health Nutr..

[27] Lee S.H., Kim S.M., Park H.S., Choi K.M., Cho G.J., Ko B.J., Kim J.H. (2013). Serum 25-hydroxyvitamin D levels, obesity and the metabolic syndrome among Korean children. Nutr. Metab. Cardiovasc. Dis..

[28] Ross R., Neeland I.J., Yamashita S., Shai I., Seidell J., Magni P., Santos R.D., Arsenault B., Cuevas A., Hu F.B. (2020). Waist circumference as a vital sign in clinical practice: A Consensus Statement from the IAS and ICCR Working Group on Visceral Obesity. Nat. Rev. Endocrinol..

[29] Kim M.Y., An S., Shim Y.S., Lee H.S., Hwang J.S. (2024). Waist-height ratio and body mass index as indicators of obesity and cardiometabolic risk in Korean children and adolescents. Ann. Pediatr. Endocrinol. Metab..

[30] Qorbani M., Heidari-Beni M., Ejtahed H.S., Shafiee G., Goodarzi F., Tamehri Zadeh S.S., Khademian M., Mohammadian Khonsari N., Motlagh M.E., Asayesh H. (2021). Association of vitamin D status and cardio-metabolic risk factors in children and adolescents: The CASPIAN-V study. BMC Nutr..

[31] Ganji V., Zhang X., Shaikh N., Tangpricha V. (2011). Serum 25-hydroxyvitamin D concentrations are associated with prevalence of metabolic syndrome and various cardiometabolic risk factors in US children and adolescents based on assay-adjusted serum 25-hydroxyvitamin D data from NHANES 2001–2006. Am. J. Clin. Nutr..

[32] Eissa M.A., Mihalopoulos N.L., Holubkov R., Dai S., Labarthe D.R. (2016). Changes in Fasting Lipids during Puberty. J. Pediatr..

[33] Heller D.A., de Faire U., Pedersen N.L., Dahlén G., McClearn G.E. (1993). Genetic and environmental influences on serum lipid levels in twins. N. Engl. J. Med..

[34] Hajhashemy Z., Shahdadian F., Moslemi E., Mirenayat F.S., Saneei P. (2021). Serum vitamin D levels in relation to metabolic syndrome: A systematic review and dose-response meta-analysis of epidemiologic studies. Obes. Rev..

[35] Li Y., Ma Z., Li Y., Xiong T., Zhang Z., Kong B., Lu W., Zhao X., Zheng R., Tang Y. (2025). Cross-sectional and longitudinal associations between serum vitamin D and continuous metabolic syndrome score among children and adolescents: Roles of levels of inflammation in peripheral blood. Nutr. Metab..

[36] Kim J., Park J., So W.Y. (2022). Association between Blood Vitamin D Levels and Regular Physical Activity in Korean Adolescents. Healthcare.

[37] Zhu L., Yao D. (2025). Research advances in children’s sleep and vitamin D levels. Ann. Pediatr. Endocrinol. Metab..

