# Supplementary material for: Associations Between Serum 25-Hydroxyvitamin D Levels and Metabolic Syndrome Among Korean Adolescents: Based on the Korea National Health and Nutrition Examination Survey in 2022–2023
Source: Nutrients. 2026 Jan 22;18(2):360. doi: 10.3390/nu18020360 (PMC12845353; doi:10.3390/nu18020360)
Supplement: Supplementary file 1 [file nutrients-18-00360-s001.zip › nutrients-4089219-supplementary.pdf]

Supplementary Table S1. Weighted sex-specific characteristics of study participants with corresponding p values and unadjusted regression coefficients with p values for linear trends across serum 25-hydroxyvitamin D [25(OH)D] quartiles assessed using complex samples regression.

				Serum 25(OH)D (ng/mL)				Unadjusted	
	Male	Female	P value	Q1 (0-13.43)	Q2 (13.43-17.88)	Q3 (17.88-22.84)	Q4 (≥22.84)	B (95% CI)	P for trend
N	457	423		205	211	229	235		
Age (years)	14.14±0.14	13.90±0.14	0.220	15.02±0.19	14.19±0.20	13.61±0.19	13.27±2.10	-0.58 (-0.77, -0.40)	<0.001
Height (cm)	166.29±0.67	157.66±0.42	<0.001	164.42±0.81	162.39±0.86	161.54±0.87	160.11±1.08	-1.38 (-2.22, -0.53)	0.001
Height SDS	0.82±0.06	0.57±0.06	0.002	0.66±0.09	0.71±0.08	0.76±0.07	0.66±0.10	0.01 (-0.07, 0.08)	0.873
Weight (kg)	61.78±1.03	51.52±0.73	<0.001	60.39±1.28	57.83±1.04	55.84±1.62	53.19±1.34	-2.36 (-3.50, -1.22)	<0.001
Weight SDS	0.80±0.09	0.49±0.07	0.009	0.79±0.13	0.76±0.09	0.62±0.14	0.44±0.09	-0.12 (-0.22, -0.02)	0.017
BMI (kg/m^2)	22.05±0.28	20.56±0.23	<0.001	22.15±0.36	21.76±0.30	21.03±0.43	20.37±0.30	-0.61 (-0.90, -0.31)	<0.001
BMI SDS	0.48±0.09	0.24±0.08	0.047	0.53±0.13	0.50±0.10	0.31±0.14	0.13±0.09	-0.14 (-0.24, -0.04)	0.008
WC (cm)	75.34±0.74	68.06±0.56	<0.001	73.69±0.91	73.15±0.82	70.67±1.13	69.49±0.94	-1.51 (-2.32, -0.69)	<0.001
WC/Ht ratio	0.45±0.00	0.43±0.00	<0.001	0.45±0.01	0.45±0.00	0.44±0.01	0.43±0.00	-0.01 (-0.01, -0.00)	0.017
Alcohol (%)	27.4	17.3	0.012	29.4	24.5	18.1	16.2	-0.27 (-0.47, -0.07)	0.008
SBP (mmHg)	111.33±0.56	105.03±0.47	<0.001	108.46±0.72	108.40±0.68	108.59±0.84	107.43±0.77	-0.29 (-0.90, 0.31)	0.340
DBP (mmHg)	65.34±0.43	64.79±0.36	0.278	66.20±0.48	64.89±0.58	64.77±0.56	64.42±0.58	-0.55 (-1.01, -0.09)	0.019
Fasting glucose (mg/dL)	92.17±0.44	90.57±0.52	0.010	89.90±0.59	92.00±0.83	91.70±0.61	91.98±0.87	0.59 (-0.05, 1.24)	0.070
Total cholesterol (mg/dL)	153.69±0.65	163.96±1.50	0.031	161.70±2.32	163.92±1.97	160.88±1.90	160.50±2.05	-0.67 (-2.56, 1.23)	0.491
Triglyceride (mg/dL)	95.72±1.33	86.41±2.40	0.216	90.08±4.37	88.98±3.19	90.97±3.41	88.60±7.40	-0.84 (-5.88, 4.19)	0.742
HDL (mg/dL)	53.69±0.65	58.37±0.78	<0.001	56.13±0.95	55.54±1.06	55.34±1.01	56.79±1.05	0.18 (-0.66, 1.02)	0.676
LDL (mg/dL)	95.72±1.33	97.08±1.37	0.462	95.84±2.18	99.48±1.64	96.22±1.80	93.99±1.85	-0.89 (-2.61, 0.84)	0.313
Sleep duration (hours)	7.43±0.06	7.32±0.07	0.258	7.20±0.10	7.37±0.08	7.51±0.09	7.48±0.10	0.10 (0.01, 0.20)	0.026
Sedentary time (hours)	11.39±0.17	11.69±0.18	0.243	11.95±0.22	11.34±0.23	11.36±0.25	11.42±0.32	-0.17 (-0.41, 0.07)	0.165
Physical activity (%)	51.1	37.2	0.003	40.3	42.6	47.6	48.8	0.12 (-0.03, 0.27)	0.105
Muscle exercise (%)	62.1	27.2	<0.001	35.8	44.5	44.6	60.5	0.30 (0.14, 0.45)	<0.001

https://doi.org/10.3390/nu18020360

All continuous variables are presented as weighted means ± standard deviations, and categorical variables are presented as weighted percentages (%). Abbreviations: 25(OH)D, 25-hydroxyvitamin D; Q1-Q4, quartiles of serum 25-hydroxyvitamin D; B, regression coefficient; CI, confidence interval; SDS, standard deviation score; BMI, body mass index; WC, waist circumference; Ht, height; SBP, systolic blood pressure; DBP, diastolic blood pressure; HDL, high-density lipoprotein cholesterol; LDL, low-density lipoprotein cholesterol.

https://doi.org/10.3390/nu18020360
